# Early and long-term responses of intestinal microbiota and metabolites to ^131^I treatment in differentiated thyroid cancer patients

**DOI:** 10.1186/s12916-024-03528-3

**Published:** 2024-07-18

**Authors:** Ganghua Lu, Dingwei Gao, Yixian Liu, Xiaqing Yu, Wen Jiang, Zhongwei Lv

**Affiliations:** 1 Clinical Nuclear Medicine Center, Imaging Clinical Medical Center, Institute of Nuclear Medicine, Institute of Clinical Mass Spectrometry Applied Research Center, Department of Nuclear Medicine, Shanghai Tenth People’s Hospital, School of Medicine, Tongji University, Shanghai, 200072 China; 2 Department of Gynecology and Obstetrics, Shanghai Tenth People’s Hospital, School of Medicine, Tongji University, Shanghai, 200072 China; 3 Department of Nuclear Medicine, Shanghai East Hospital, School of Medicine, Tongji University, Shanghai, 200092 China; 4 Shanghai Public Health Clinical Center, Fudan University, Shanghai, 200003 China

**Keywords:** ^131^I therapy, Gut microbiota, Fecal metabolites, Linoleic acid

## Abstract

**Background:**

Multiple high doses of ^131^I therapy in patients with differentiated thyroid cancer (DTC) might disrupt the balance of gut microbiota and metabolites. This study aimed to investigate the alterations of intestinal bacteria and metabolism over two courses of ^131^I therapy, explore the interactions, and construct diagnostic models reflecting enteric microecology based on ^131^I therapy.

**Methods:**

A total of 81 patients were recruited for the first ^131^I therapy (^131^I-1st), among whom 16 received a second course (^131^I-2nd) after half a year. Fecal samples were collected 1 day before (Pre-^131^I-1st/2nd) and 3 days after (Post-^131^I-1st/2nd) ^131^I therapy for microbiome (16S rRNA gene sequencing) and metabolomic (LC–MS/MS) analyses.

**Results:**

A total of six microbial genera and 11 fecal metabolites enriched in three pathways were identified to show significant differences between Pre-^131^I-1st and other groups throughout the two courses of ^131^I treatment. In the Post-^131^I-1st group, the beneficial bacteria *Bifidobacterium*,* Lachnoclostridium*,* uncultured_bacterium_f_Lachnospiraceae*, and *Lachnospiraceae_UCG004* were abundant and the radiation-sensitive pathways of linoleic acid (LA), arachidonic acid, and tryptophan metabolism were inhibited compared with the Pre-^131^I-1st group. Compared with the Pre-^131^I-1st group, the Pre-^131^I-2nd group exhibited a reduced diversity of flora and differentially expressed metabolites, with a low abundance of beneficial bacteria and dysregulated radiation-sensitive pathways. However, less significant differences in microbiota and metabolites were found between the Pre/Post-^131^I-2nd groups compared with those between the Pre/Post-^131^I-1st groups. A complex co-occurrence was observed between 6 genera and 11 metabolites, with *Lachnoclostridium*, *Lachnospiraceae_UCG004*, *Escherichia-Shigella*, and LA-related metabolites contributing the most. Furthermore, combined diagnostic models of charactered bacteria and metabolites answered well in the early, long-term, and dose-dependent responses for ^131^I therapy.

**Conclusions:**

Different stages of ^131^I therapy exert various effects on gut microecology, which play an essential role in regulating radiotoxicity and predicting the therapeutic response.

**Supplementary Information:**

The online version contains supplementary material available at 10.1186/s12916-024-03528-3.

## Background

As the most common endocrine malignancy, thyroid cancer (TC) has an increasing annual global incidence, with differentiated thyroid cancer (DTC) accounting for approximately 90% of the cases [1]. Most patients with DTC exhibit positive outcomes after standard treatments, including surgery, ^131^I, and levothyroxine replacement for suppression of thyroid hormones. However, some patients require multiple ^131^I therapies with higher doses due to the residual thyroid tissue and local or distant metastatic mass, which increases the chances of adverse reactions in the hematopoietic, gastrointestinal, and even marrow systems [2, 3]. Nonetheless, few noninvasive tests are available to investigate the early and long-term responses of DTC patients under ^131^I therapy.

Since ^131^I is administered orally, it accumulates in the gastrointestinal tract and may affect gut microbes [4]. Until now, many studies have reported that ionizing radiation (IR) affects the structure, function, and species of the gut microbiota and its metabolites by triggering dysbiosis, further exacerbating damage induced by IR [5–8]. On the contrary, beneficial bacteria and favorable metabolites could rebuild the IR-injured gut microbial structure via anti-inflammatory and anti-oxidant mechanisms [6, 9, 10]. In addition, previous studies also revealed a significant difference in gut microbes and metabolites between DTC patients and health controls, with the imbalanced composition of microbiota and homeostasis of lipid metabolism possibly accelerating the progression of thyroid cancer [11, 12]. Interestingly, profiles of gut microbiota could also predict the treatment effect of ^131^I therapy in DTC patients [4]. All these findings indicated that the gut microbiome and its related metabolites were closely related to DTC and ^131^I therapy, highlighting its importance in the prevention of radiotoxicity and risk factors of DTC progression.

Nevertheless, the functional alterations in enteric microbiota and their radiation-associated metabolites at different stages of ^131^I treatment in DTC remain unclear. Hence, gut 16S-rDNA sequencing and metabolome analyses were performed in a cohort of patients with DTC over two courses to validate ^131^I-related changes in the microbial and metabolic signatures of DTC patients.

## Methods

### Study subjects and design

The study enrolled 81 patients with DTC undertaking the first ^131^I therapy (^131^I-1st) and 16 follow-ups among them after half a year undertaking the second ^131^I therapy (^131^I-2nd) from the Department of Nuclear Medicine, Shanghai Tenth People’s Hospital, between March 2021 and March 2022 (Table 1). The diagnosis of DTC was based on the following criteria: (i) clinically diagnosed with DTC within 6 months [13], (ii) age between 18 and 75 years, female (nonpregnant) or male without history of other malignancies, and (iii) receiving ^131^I treatment. The following patients were also excluded: (i) patients confirmed not to have thyroid cancer on postoperative pathology, (ii) subjects suffering from a gastrointestinal disease or any other severe physical or mental disease, and (iii) subjects with a history of probiotics, orally ingested antibiotics, or any other similar drug within 2 months before fecal sampling. Blood routine examination, thyroid function, and biochemical and inflammatory indicators in the blood samples were measured (Table 1). The study was carried out following the Declaration of Helsinki principles and approved by the Ethics Committee of the Shanghai Tenth People’s Hospital (No. SHSY-IEC-5.0/22K13/P01). Informed, written consent was obtained from all patients.

**Table 1:** Baseline characteristics of patients in ^131^I-1st and ^131^I-2nd groups

Variables	^131^I-1st (*n* = 81)	^131^I-2nd (*n* = 16)	*P *value
Gender, male (%)	23 (28)	7 (46)	0.246
Age, years	47 ± 15	49 ± 11	0.647
BMI, kg/m^2^	25.30 ± 4.25	26.09 ± 3.45	0.488
^131^I, mCi	100 (100–120)	135 (100–195)	0.047*
Clinical stage, II-IV (%)	9 (11)	8 (50)	0.001*
Thyroid function			
TSH, mIU/L	106.00 ± 33.73	104.87 ± 36.93	0.904
Tg, ng/mL	38.06 ± 104.32	75.40 ± 146.88	0.240
TgAb, ≥ 100.00 IU/mL (%)	20 (26)	2 (12)	0.344
Routine blood			
WBC, 10^9^/L	5.80 (4.92–6.92)	6.53 (5.52–8.35)	0.143
RBC, 10^12^/L	4.70 ± 0.44	4.62 ± 0.35	0.502
PLT, 10^9^/L	246 ± 65	226 ± 75	0.292
Hb, g/L	139 ± 18	142 ± 13	0.493
Gran, %	59.2 (53.8–69.2)	63.9 (58.6–71.0)	0.081
Lymph, %	31.8 ± 8.6	26.6 ± 8.8	0.035*
Inflammation			
TNFα, pg/mL	6.48 (5.26–8.10)	5.78 (5.06–6.48)	0.181
IL-1, ≥ 5.00 pg/mL (%)	16 (21)	3 (19)	1.000
IL-2, ≥ 200.00 u/mL (%)	20 (26)	7 (44)	0.224
IL-6, ≥ 2.00 pg/mL (%)	42 (55)	10 (62)	0.594
CRP, ≥ 3.13 mg/L (%)	11 (14)	1 (6)	0.684
Liver function			
TBI, μmol/L	13.8 ± 6.0	13.5 ± 5.1	0.887
DBI, μmol/L	4.2 ± 1.5	4.2 ± 1.5	0.988
TCh, mmol/L	6.41 (5.75–7.60)	6.68 (5.60–7.70)	0.612
TG, mmol/L	1.92 (1.26–2.68)	1.89 (1.10–3.20)	0.952
HDL, mmol/L	1.8 ± 0.4	1.8 ± 0.4	0.874
LDL, mmol/L	3.9 (3.2–4.7)	3.7 (3.1–4.9)	0.929

Data were presented as numbers of patients (*n*, %), mean ± standard deviations or median (interquartile range).

*Comparison between ^131^I-1st and ^131^I-2nd groups.

*BMI* body mass index, *CRP* C-reactive protein, *DBI* direct bilirubin, *DTC* differentiated thyroid cancer, *Gran* neutrophil, *Hb* hemoglobin, *HDL* high density lipoprotein, *IL-1* interleukin 1, *IL-2* interleukin 2 receptor, *IL-6* interleukin 6, *LDL* low density lipoprotein, *Lymph* lymphocyte, *PLT* platelet, *RBC* red blood cell, *TBI* total bilirubin, *TCh* total cholesterol, *TG* triglyceride, *Tg* thyroglobulin, *TgAb* thyroglobulin antibodies, *TNFα* tumor necrosis factor alpha, *TSH* thyrotropin, *WBC* white blood cell. ^131^I-1st group, patients with DTC undertaking the first ^131^I therapy; ^131^I-2nd group, patients with DTC undertaking the second ^131^I therapy.

### Collection of fecal samples

Fecal samples were collected in the morning after an overnight fast (> 8 h) 1 day before (Pre-^131^I-1st/2nd) and 3 days after (Post-^131^I-1st/2nd) ^131^I treatment. Fecal samples were snap-frozen with liquid nitrogen, collected, and stored at − 80 °C (Haier, DW-86L626, China) [14].

### Microbiomic analysis

Microbial DNA was extracted from fecal samples using a QIAamp Fast DNA stool Mini Kit (Qiagen, Cat# 51,604) and amplified with barcoded bacterial primers targeting the 16S rRNA variable region 3–4 (V3–V4): forward primer 338F:5′-ACTCCTACGGGAGGCAGCA-3′ and reverse primer 806R:5′-GGACTACHVGGGTWTCTAAT-3′ [15]. Illumina NovaSeq6000 was used for the construction of sequencing libraries and paired-end sequencing of samples. Paired-end reads were merged using FLASH v1.2.7, and tags with more than six mismatches were removed [16]. Using Trimmomatic, tags with a quality score of 20 within a sliding window of 50 bps were determined, and those shorter than 350 bps were removed [17]. A total of 172 chimeras were removed from the denoised sequences and they were clustered into operational taxonomic units (OTUs) with a 97% similarity using the USEARCH (version 10.2) program. All OTUs were assigned a taxonomy by searching the Silva databases (Release128) using QIIME.

### Untargeted metabolomic analysis

Fifty milligrams of fecal sample was weighed into an EP tube, and 1000 μL of extract solution (methanol: acetonitrile: water = 2:2:1, with isotopically labeled internal standard mixture) was added. The samples were then homogenized at 35 Hz for 4 min and sonicated for 5 min in an ice-water bath. Homogenization and sonication cycles were repeated for three times. The samples were then incubated for 1 h at − 40 °C and centrifuged at 12,000 rpm for 15 min at 4 °C. The resulting supernatant was transferred into a fresh glass vial for further analysis. A quality control sample was prepared by mixing an equal aliquot of the supernatant from all samples.

### LC–MS/MS analysis

LC–MS/MS analyses were performed using an UHPLC system (Vanquish, Thermo Fisher Scientific) with a UPLC BEH Amide column (2.1 mm × 100 mm, 1.7 μm) coupled to Q Exactive HFX mass spectrometer (Orbitrap MS, Thermo). The mobile phase consisted of 25 mmol/L ammonium acetate and 25 ammonia hydroxides in water (pH = 9.75) (A) and acetonitrile (B). The auto-sampler temperature was 4 ℃, and the injection volume was 3 μL. The QE HFX mass spectrometer was used for its ability to acquire MS/MS spectra on information-dependent acquisition (IDA) mode in the control of the acquisition software (Xcalibur, Thermo). In this mode, the acquisition software continuously evaluates the full scan MS spectrum. The ESI source conditions were set as follows: sheath gas flow rate as 30 Arb, Aux gas flow rate as 25 Arb, capillary temperature 350 ℃, full MS resolution as 60,000, MS/MS resolution as 7500, collision energy as 10/30/60 in NCE mode, spray Voltage as 3.6 kV (positive) or − 3.2 kV (negative), respectively.

### Statistical analysis

Data are presented as the mean ± standard deviation or median (interquartile range). To assess significance, the mean values of independent groups were compared using Student’s *t* test and Wilcoxon rank-sum test; categorical variables were assessed by the chi-square test as follows: *, *p* < 0.05; **, *p* < 0.01; ***, *p* < 0.001; ****, *p* < 0.0001. False discovery rate (FDR) was used to adjust *p* values in differential expressed bacteria and metabolite analyses. Statistical significance was set at *p* < 0.05.

## Results

### Clinical characteristics of the participants

The basic information of the 81 DTC patients receiving ^131^I therapy for the first time (^131^I-1st), among whom 16 received a second course (^131^I-2nd), are displayed in Table 1. Compared with the ^131^I-1st group, the ^131^I-2nd group showed a higher clinical stage (II/III/IV) and dose of ^131^I treatment, but lower percentage of lymphocytes. In contrast, gender, age, body mass index (BMI), thyroid function, inflammation, and liver function showed no significant difference between the groups.

### Features of gut bacterial community composition and structure

**Fig. 1:**
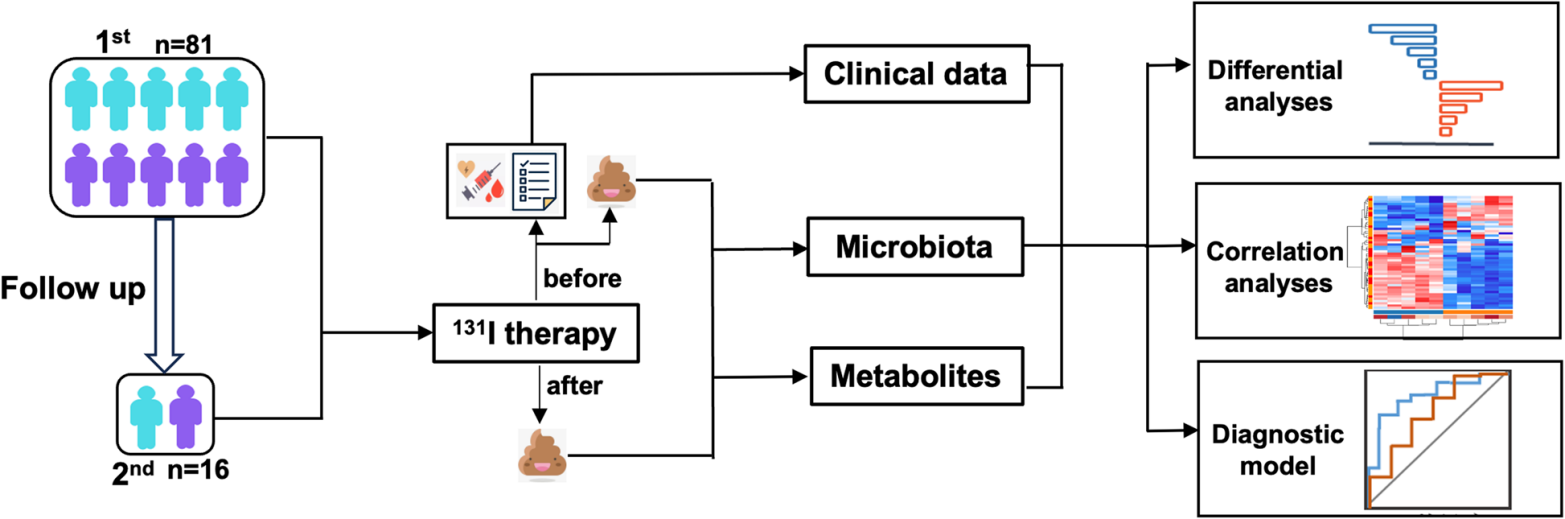
Overview of the workflow for the microbiomic and metabolomic strategy used in this study

**Fig. 2:**
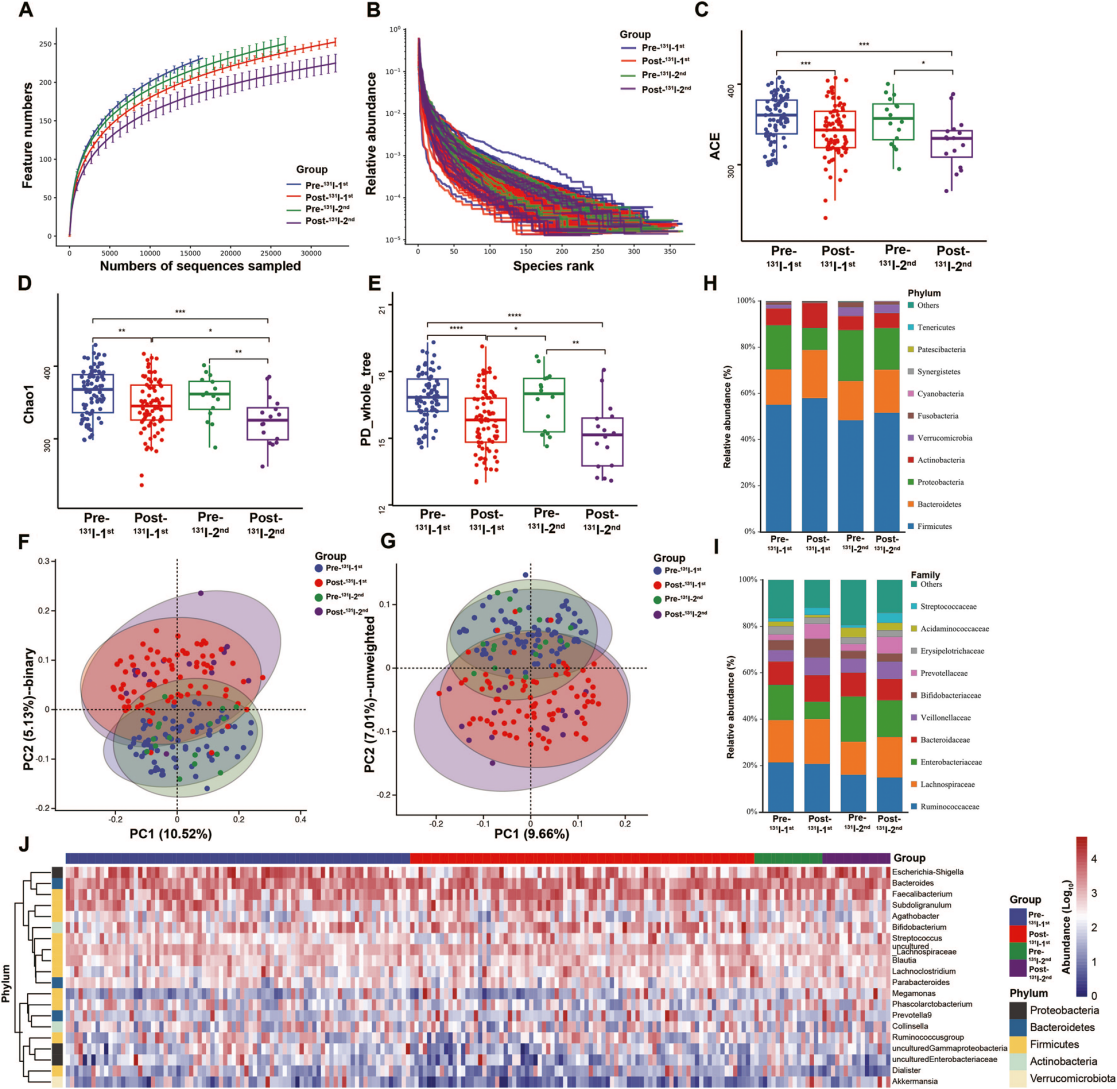
Features of gut bacterial community composition and structure. α diversity analysis in rarefaction curve (**A**), rank abundance curve (**B**), Ace (**C**), Chao1 (**D**), and PD_whole_tree (**E**) indices among four groups. β diversity analysis on principal coordinates analysis (PCoA) for binary (**F**) and unweighted distances (**G**) among four groups. Abundance distribution on Phylum (**H**) and Family (**I**) level among four groups. **J** Annotated abundance heatmap of gut microbial features of the top 20 genera based on Ward hierarchical clustering. * *p* < 0.05, ** *p* < 0.01, *** *p* < 0.001, **** *p* < 0.0001. DTC, differentiated thyroid cancer; Pre-^131^I-1st group, patients with DTC before the first ^131^I therapy; Post-^131^I-1st group, patients with DTC after the first ^131^I therapy; Pre-^131^I-2nd group, patients with DTC before the second ^131^I therapy; Post-^131^I-2nd group, patients with DTC after the second ^131^I therapy

Furthermore, microbiome analyses were performed on fecal samples from DTC patients before and after ^131^I therapy (Fig. 1). The results were processed for quality filtering, and chimeric sequences were removed, yielding an average of 58,152 sequences (derived from 67,859 raw reads) for abundance and diversity analyses. The rarefaction curve revealed great species richness and evenness (Fig. 2A) and the rank abundance curve (Fig. 2B) confirmed the thoroughness of the sample collection. The α diversity of Ace, Chao1, PD_whole_tree (Fig. 2C-E), Simpson, and Shannon (Additional file 1: Fig.S1A, B) indices were significantly decreased in the Post-^131^I-1st group compared to the Pre-^131^I-1st group and in the Post-^131^I-2nd group compared to the Pre-^131^I-2nd group. The Pre-^131^I-2nd group demonstrated a small reduction compared to the Pre-^131^I-1st group. In addition, principal coordinate analysis (PCoA) based on binary and unweighted distances (Fig. 2F, G) revealed significant differences between the Pre-^131^I-1st/2nd groups and Post-^131^I-1st/2nd groups.

Moreover, remarkable differences were observed in all groups from the Phylum to Genus levels (Fig. 2H, I). At the Phylum level, the relative abundance of *Firmicutes* was elevated in the Post-^131^I-1st/2nd groups compared to the Pre-^131^I-1st/2nd groups, which was inconsistent with *Proteobacteria* (Fig. 2H) and the ratio of *Firmicutes*/*Bacteroidetes* (F/B, Additional file 1: Fig.S1C). At the Family level, *Enterobacteriaceae* decreased in the Post-^131^I-1st group compared with Pre-^131^I-1st group, whereas *Lachnospiraceae* and *Bifidobacteriaceae* were enriched in the Post-^131^I-1st group (Fig. 2I). However, the differences between the Pre-and Post-^131^I-2nd groups were not considerable. A broad overview of the taxonomic data at the Genus level of all samples is shown in Fig. 2J.

### Alterations in microbial genus compositions at different stages of DTC with ^131^I therapy

**Fig. 3:**
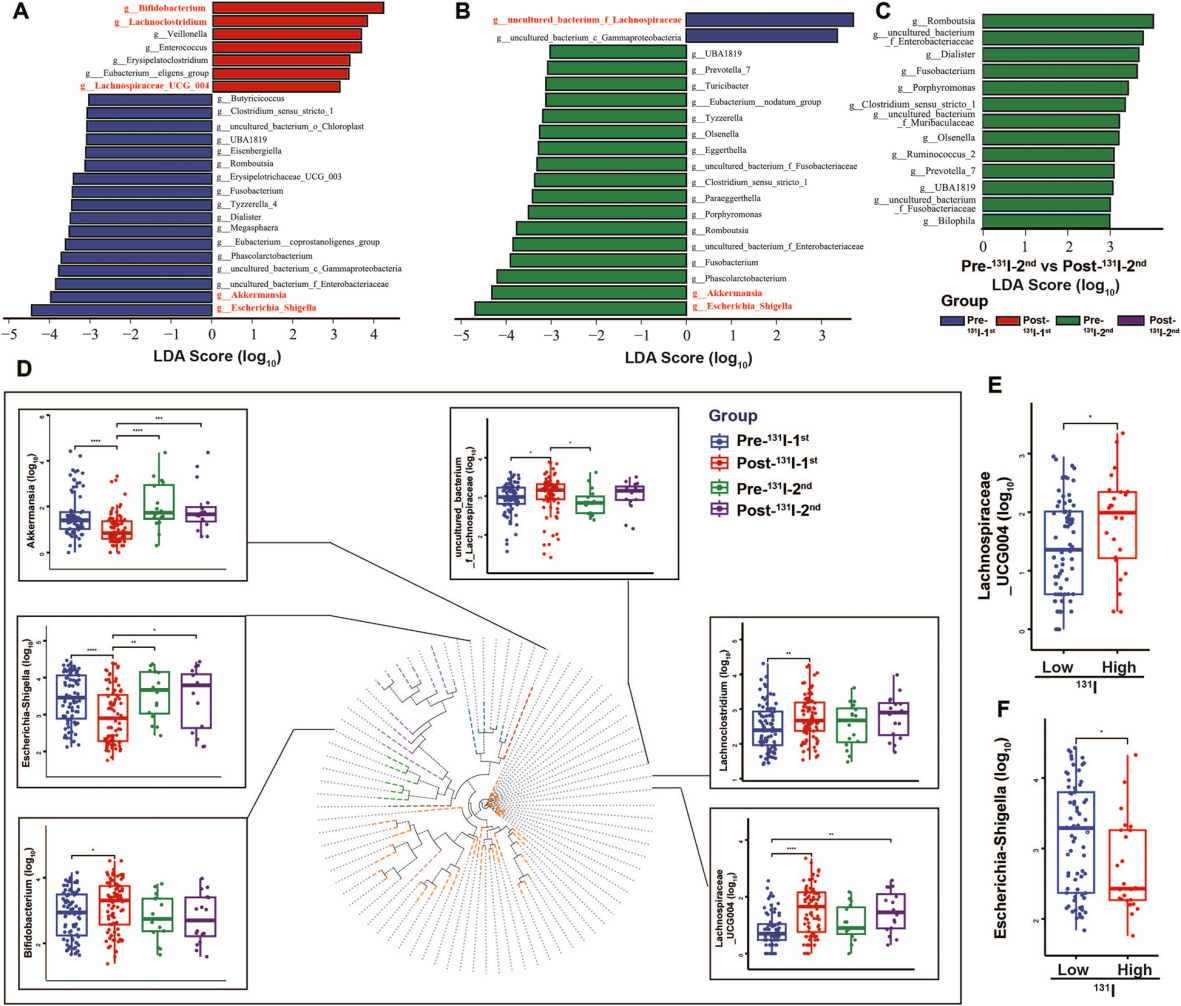
Alterations in microbial genus compositions at different stages of DTC with ^131^I therapy

To investigate the difference in bacterial composition among the 4 groups, linear discriminate analysis effect size (LEfSe) analysis (Fig. 3A–C) and the Wilcoxon rank-sum test (Additional file 2: Fig.S2A-C) were conducted. According to the above results, two altered patterns were identified on the genus level. The first pattern showed an increased abundance, while the second pattern presented a decreased abundance in the Post-^131^I-1st/2nd groups compared to the Pre-^131^I-1st/2nd groups. The first pattern included four genera, including *Bifidobacterium*,* Lachnoclostridium*,* uncultured_bacterium_f_Lachnospiraceae*, and *Lachnospiraceae_UCG004* (Fig. 3D). The second pattern included two genera, namely *Akkermansia* and *Escherichia-Shigella* (Fig. 3D). Intriguingly, *uncultured_bacterium_f_Lachnospiraceae* in the first pattern showed a lower abundance in the Pre-^131^I-2nd group compared to the Pre-^131^I-1st group, displaying an opposite trend in pattern2 (Fig. 3B). Similar with the results at the Family level, the differences between the Pre- and Post-^131^I-2nd groups were not obvious (Fig. 2H; Fig. 3D). Additionally, the Post-^131^I-1st/2nd groups were integrated and divided into the ^131^I-high dose (for metastatic diseases, *n* = 24, ≥ 150 mCi) and ^131^I-low dose (for thyroid remnants or adjuvant therapy, *n* = 73, < 150 mCi) groups [18, 19]. Surprisingly, the abundance of *Lachnospiraceae_UCG004* was significantly improved in the high-dose group (Fig. 3E), showing reversed results to *Escherichia-Shigella* (Fig. 3F) and *Akkermansia* (Additional file 2: Fig.S2D).

Linear discriminate analysis (LDA) effect size (LEfSe) revealing differential microbiota on the genus level between Post- and Pre-^131^I-1st (**A**), Pre-^131^I-2nd and Pre-^131^I-1st (**B**), Post- and Pre-^131^I-2nd (**C**), charactered genera marked with red. **D** Taxonomic analysis showed six microbial genera were changed among four groups. Abundance of *g_Lachnospiraceae_UCG004* (**E**) and *g_Escherichia-Shigella* (**F**) between low (< 150 mCi) and high (≥ 150 mCi) dose of ^131^I therapy. * *p* < 0.05, ** *p* < 0.01, *** *p* < 0.001, **** *p* < 0.0001. DTC, differentiated thyroid cancer; Pre-^131^I-1st group, patients with DTC before the first ^131^I therapy; Post-^131^I-1st group, patients with DTC after the first ^131^I therapy; Pre-^131^I-2nd group, patients with DTC before the second ^131^I therapy; Post-^131^I-2nd group, patients with DTC after the second ^131^I therapy.

### Fecal metabolomic profiles based on ^131^I treatment progress

**Fig. 4:**
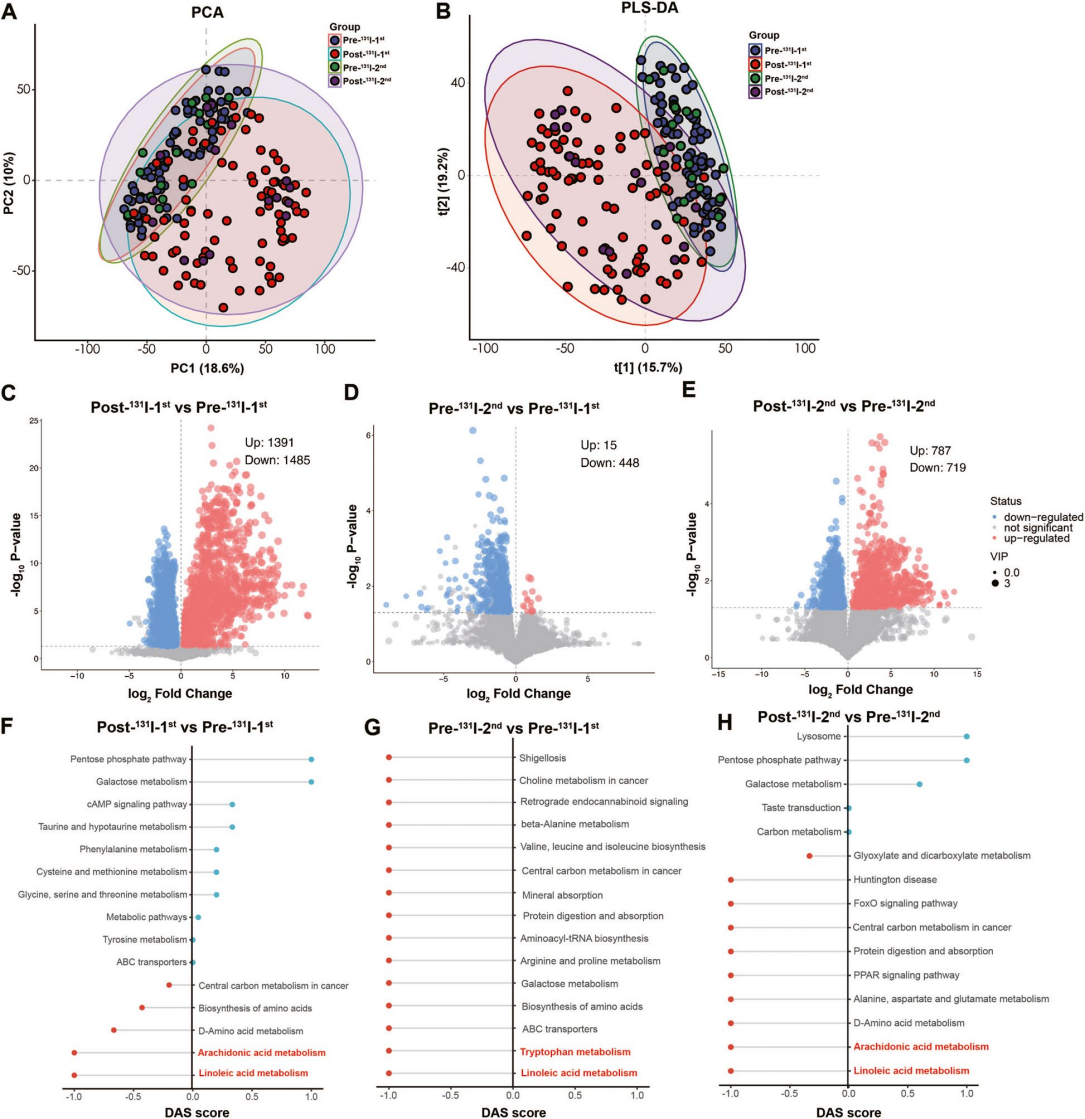
Fecal metabolomic profiles in accordance with ^131^I treatment progress. Principle components analysis (PCA) (**A**) and partial least square regressions-discriminant analysis (PLS-DA) (**B**) results among four groups. Volcano plot of differential metabolites (fold change ≤ 0.5 or > 2) between Post- and Pre-^131^I-1st (**C**), Pre-^131^I-2nd and Pre-^131^I-1st (**D**), Post-and Pre-^131^I-2nd (**E**). Differential abundance scores (DAS) of KEGG pathways between Post- and Pre-^131^I-1st (**F**), Pre-^131^I-2nd and Pre-^131^I-1st (**G**), Post- and Pre-^131^I-2nd (**H**), radiation-sensitive metabolic pathways marked red. DTC, differentiated thyroid cancer; Pre-^131^I-1st group, patients with DTC before the first ^131^I therapy; Post-^131^I-1st group, patients with DTC after the first ^131^I therapy; Pre-^131^I-2nd group, patients with DTC before the second ^131^I therapy; Post-^131^I-2nd group, patients with DTC after the second ^131^I therapy

Next, the metabolome of the enteric flora was evaluated in DTC patients to reveal the alterations in metabolites during continued ^131^I therapy (Fig. 1). The score plots of principal components analysis (PCA) and partial least square regressions-discriminant analysis (PLS-DA) clearly separated the Pre-^131^I-1st/2nd groups from Post-^131^I-1st/2nd groups (Fig. 4A, B). These findings revealed an obvious metabolite variability across different stages of ^131^I treatment. According to the results of pairwise contrasts by volcano plots, a greater number of downregulated differential metabolites were observed in the Post-^131^I-1st group compared to the Pre-^131^I-1st group (Fig. 4C). In addition, fewer metabolites were upregulated in the Pre-^131^I-2nd group compared to the Pre-^131^I-1st group (Fig. 4D); however, no distinct difference was found between the Pre- and Post-^131^I-2nd groups (Fig. 4E). Kyoto Encyclopedia of Genes and Genomes (KEGG) pathway enrichment analysis was conducted for all differential metabolites, further exploring their biological function (Fig. 4F–H). Notably, the two essential metabolic pathways involved in LA metabolism and arachidonic acid (ARA) metabolism were strongly downregulated in the Post-^131^I-1st/2nd groups compared to the Pre-^131^I-1st/2nd groups (Fig. 4F, H). The pathways of LA and tryptophan metabolism were downregulated in the Pre-^131^I-2nd group compared to the Pre-^131^I-1st group (Fig. 4G).

**Fig. 5:**
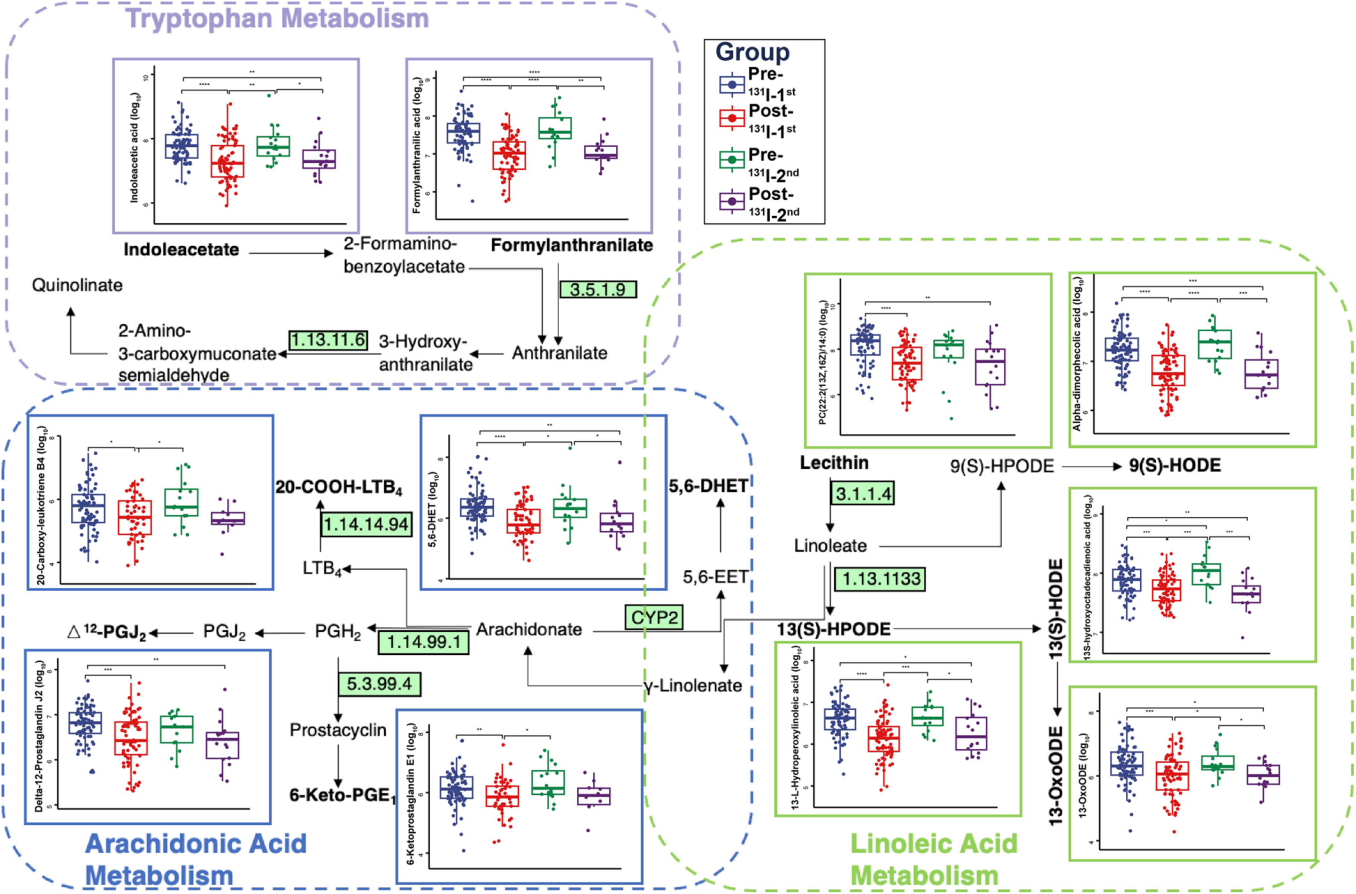
Changes of gut metabolites across the ^131^I therapy summarized in KEGG pathways. ^131^I therapy-related changes of gut metabolites summarized in radiation-sensitive metabolic pathways among four groups according to KEGG pathway reference map. * *p* < 0.05, ** *p* < 0.01, *** *p* < 0.001, **** *p* < 0.0001. DTC, differentiated thyroid cancer; Pre-^131^I-1st group, patients with DTC before the first ^131^I therapy; Post-^131^I-1st group, patients with DTC after the first ^131^I therapy; Pre-^131^I-2nd group, patients with DTC before the second ^131^I therapy; Post-^131^I-2nd group, patients with DTC after the second ^131^I therapy

To investigate the influence of the differential metabolites in these three metabolic pathways (radiation-sensitive metabolic pathways), reaction pathways were manually constructed using the KEGG pathway reference map (Fig. 5). The results revealed that five metabolites (alpha-dimorphecolic acid, 13-OxoODE, 13-L-Hydroperoxylinoleic acid, PC(22:2(13Z,16Z)/14:0), and 13S-hydroxyoctadecadienoic acid) derived from the LA metabolism pathway, four metabolites (5,6-DHET, 20-Carboxy-leukotriene B4, 6-Ketoprostaglandin E1, delta-12-Prostaglandin J2) derived from the ARA metabolism pathway, and two metabolites (indoleacetic acid, formylanthranilic acid) derived from the tryptophan metabolism pathway were consistent with their pathways’ regulation by ^131^I therapy. Meanwhile, a close metabolic association was found between the LA and ARA metabolism pathways (Fig. 5). Our previous study and other research have reported that the administration of metabolites in ARA and tryptophan metabolism pathways could exert an anti-radiotoxicity effect [6, 9, 20, 21]. Additionally, anti-inflammatory and antioxidative effects have also been linked to intestinal LA metabolism pathways [22, 23]. Our study also preliminarily revealed that the supplementation of LA in mice undergoing ^131^I therapy could provide some protection against radiation toxicity (Additional file 3: Fig.S3A-S3F; Additional file 4: Supplemental Methods). Therefore, metabolites in these radiation-sensitive metabolic pathways might play a crucial role in radiation protection.

### Disrupted gut microbiota interactions with fecal metabolism and clinical factors associated with DTC patients under ^131^I therapy

**Fig. 6:**
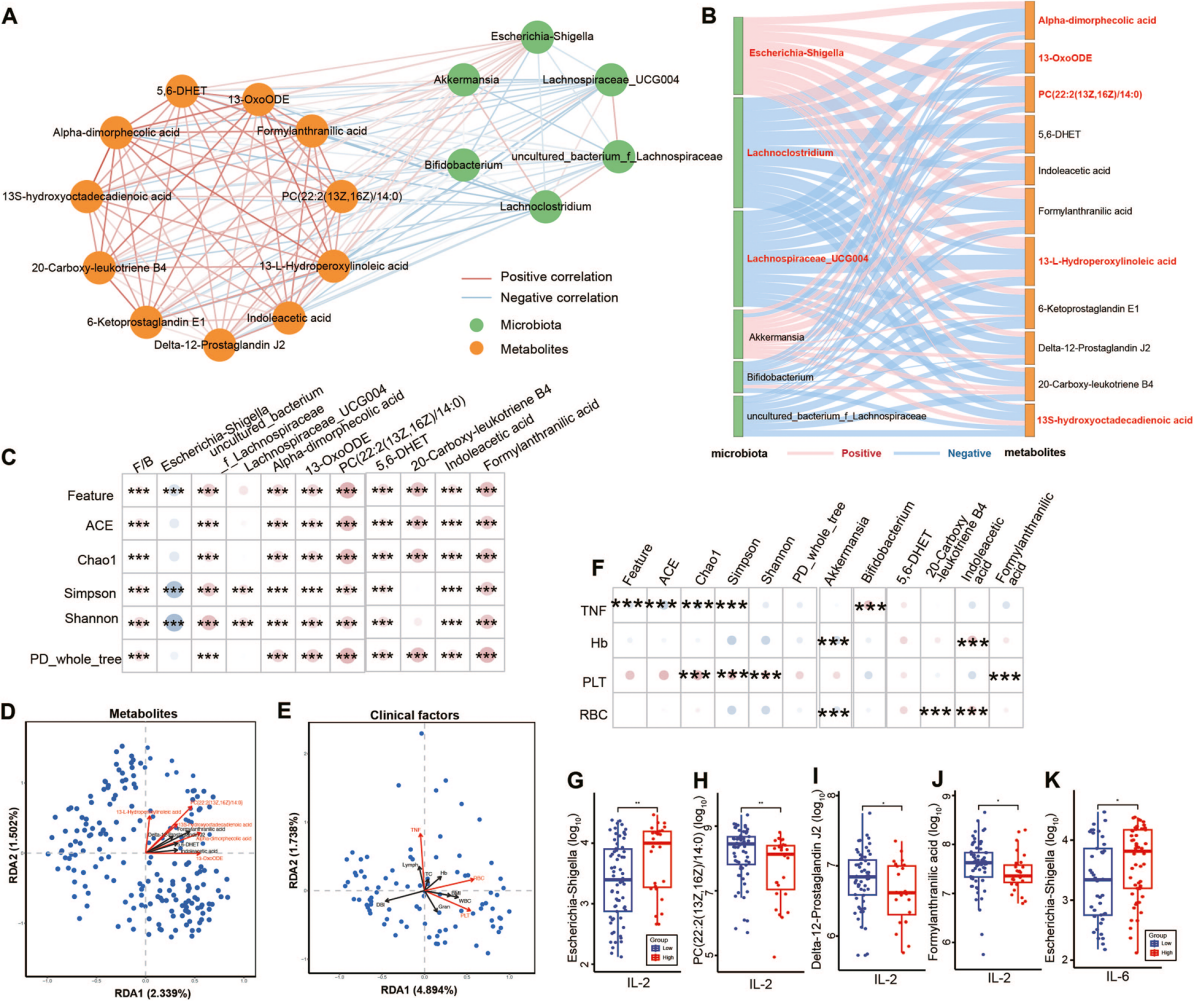
Disrupted gut microbiota interactions with fecal metabolism and clinical factors associated with DTC patients under ^131^I therapy. **A** Integrated network of ^131^I therapy -related microbial genera and fecal metabolites. Networks were constructed using NAMAP with Spearman’s rank correlations. **B** The SANKEY diagrams show associations between ^131^I therapy-related microbial genera and fecal metabolites. The most significant factors for the associations marked red. **C** Spearman correlation analyses between α diversity indices and ^131^I therapy-related microbial genera and fecal metabolites. Redundancy analysis (RDA) analysis of metabolites (**D**) and clinical factors (**E**) on the Genus level, red marked factors contributed most to sample distribution. **F** Spearman correlation analyses between clinical factors important in RDA and ^131^I therapy-related microbial genera and fecal metabolites. Abundance of *Escherichia-Shigella* (**G**), and levels of PC(22:2(13Z,16Z)/14:0) (**H**), Delta-12-Prostaglandin J2 (**I**), and Formylanthranilic acid (**J**) between low and high levels of interleukin 2 receptor (IL-2). **K** Abundance of *Escherichia-Shigella* between low and high levels of interleukin 6 (IL-6). * *p* < 0.05, ** *p* < 0.01, *** *p* < 0.001

An integrated network analysis was performed according to the microbial and metabolic data to identify the possible mechanisms between differential intestinal flora and metabolites in ^131^I therapy (Fig. 6A, B; Additional file 5: Fig.S4A-C). Consequently, an elaborate co-existence pattern and a robust connection were found between ^131^I therapy progress-related microbes and metabolites from the three radiation-sensitive metabolic pathways (Fig. 6A). Meanwhile, *Lachnoclostridium*, *Lachnospiraceae_UCG004*, and *Escherichia-Shigella* counts were closely correlated with fecal metabolites in the LA metabolism pathways (Fig. 6B), which were recognized as main contributors in this integrated network, suggesting their crucial roles in the different phases of ^131^I therapy. Interestingly, *Bifidobacterium*, *Lachnoclostridium*, *uncultured_bacterium_f_Lachnospiraceae*, and *Lachnospiraceae_UCG004* were negatively associated with the pathways of LA, ARA, and tryptophan metabolism, with a converse trend in *Akkermansia* and *Escherichia-Shigella* (Fig. 6A, B). Furthermore, the α diversity of all the indices was strongly negatively correlated with *Escherichia-Shigella*, but positively correlated with F/B, *uncultured_bacterium_f_Lachnospiraceae*, *Lachnospiraceae_UCG004*, and metabolites in 3 pathways (Fig. 6C). Moreover, the redundancy analysis was performed to verify the impact of metabolites (Fig. 6D) and clinical factors (Fig. 6E) on the bacteria sample distribution. The results indicated that metabolites from the LA metabolism pathway, tumor necrosis factor-alpha (TNFα), red blood cells (RBCs), and platelets (PLTs) contributed the most to the sample distribution. Moreover, these clinical factors (Fig. 6F) and other inflammation indicators (Fig. 6G–K) showed a high correlation with the identified bacteria and metabolites.

### Predictive models for response to ^131^I therapy based on microbiome and metabolism

**Fig. 7:**
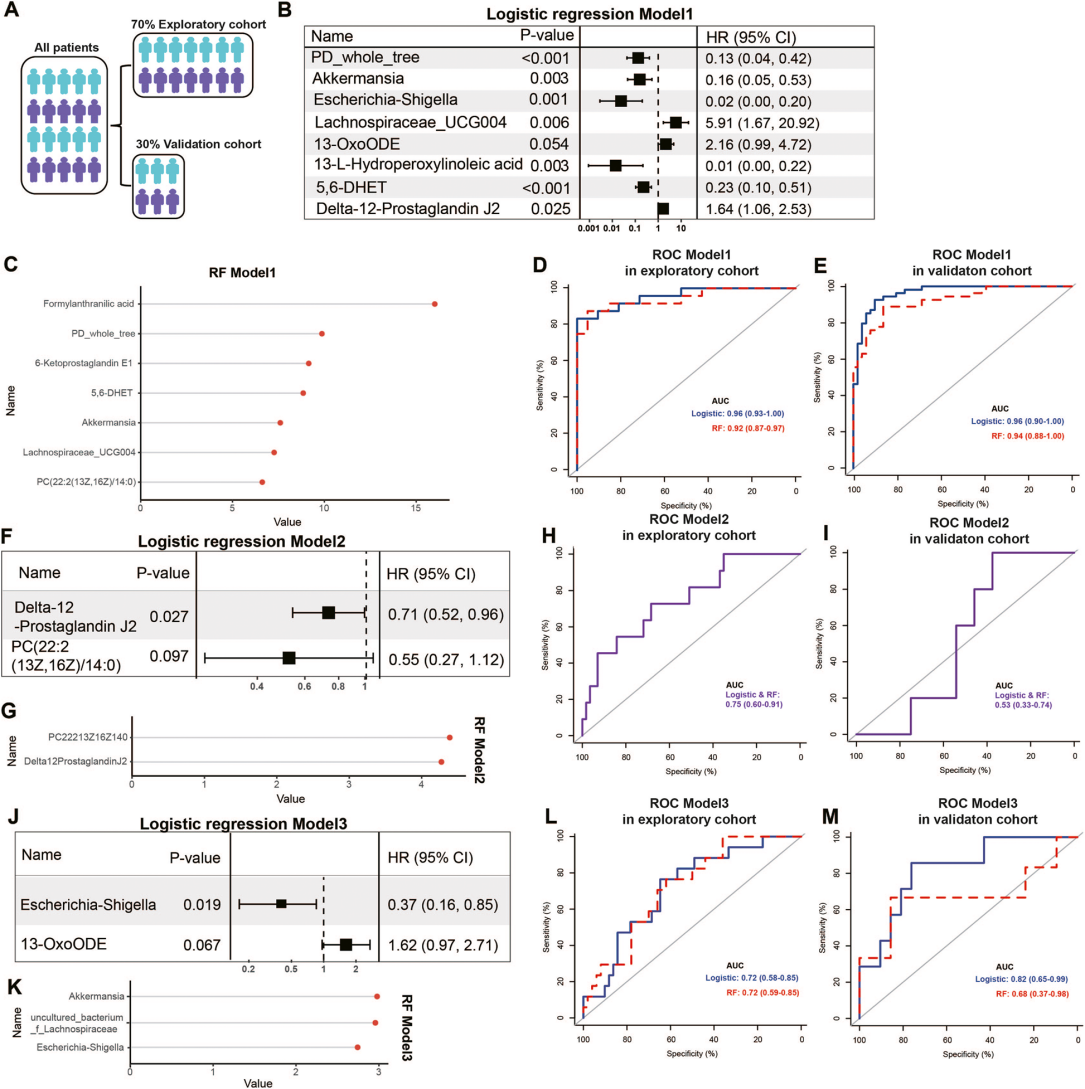
Predictive models combined with microbiome and metabolism for response to ^131^I therapy. **A** Schematic of sample distribution to exploratory cohort and validation cohort. **B** Eight factors were selected by the forward stepwise logistic regression in discriminations between Post- and Pre-^131^I-1st groups (Model1). **C** The importance ranking of the top seven factors in random forest model (RF) of Model1. Receiver operating characteristic (ROC) curves in exploration set (**D**) and validation set (**E**) of Model1. **F** Two factors were selected by the forward stepwise logistic regression in discriminations between Pre-^131^I-2nd and Pre-^131^I-1st groups (Model2). **G** The importance ranking of the top two factors in RF of Model2. ROC curves in exploration set (**H**) and validation set (**I**) of Model2. **J** Two factors were selected by the forward stepwise logistic regression in discriminations between high (≥ 150 mCi) and low (< 150 mCi) dose of ^131^I therapy groups (Model3). **K** The importance ranking of the top three factors in RF of Model3. ROC curves in exploration set (**L**) and validation set (**M**) of Model3. DTC, differentiated thyroid cancer; HR, hazard ratio; Pre-^131^I-1st group, patients with DTC before the first ^131^I therapy; Post-^131^I-1st group, patients with DTC after the first ^131^I therapy; Pre-^131^I-2nd group, patients with DTC before the second ^131^I therapy

Classifiers of logistic regression and random forest (RF) were constructed to determine the non-invasive diagnostic markers based on gut microbial and metabolomic factors. The exploratory cohort (70%) was randomly generated from the two compared groups in the corresponding model (Fig. 7A). Model1 consisted of 7–8 factors in two classifiers and was constructed to distinguish the Post-^131^I-1st from the Pre-^131^I-1st group (Fig. 7B, C; Additional file 5: Fig.S5A), achieving a total area under the curve (AUC) of 0.96 (logistic) and 0.92 (RF) (Fig. 7D); this model was further validated (*Z* > 0.05, Fig. 7E). Over half of the common factors were found between two classifiers in Model1, including PD_whole_tree in α diversity, *Akkermansia*, *Lachnospiraceae_UCG004*, and 5,6-DHET (Fig. 7B, C). These results indicated significant alterations in the gut microenvironment after ^131^I-1st. In contrast, Model2 consisted of PC(22:2(13Z,16Z)/14:0) and delta-12-Prostaglandin J2 in logistic and RF classifiers, and was constructed to discriminate between the Pre-^131^I-2nd and Pre-^131^I-1st groups (Fig. 7F, G; Additional file 6: Fig.S5B). This model achieved a total AUC of 0.75 in the two classifiers (Fig. 7H) and showed good validation (*Z* > 0.05, Fig. 7I). Despite the small difference in clinical data between the two groups (Table 1), the changes in fecal metabolites in DTC patients undergoing ^131^I therapy showed larger changes. Furthermore, Model3 consisted of 2–3 factors in logistic and RF classifiers and was constructed to discriminate the ^131^I-high dose group from the ^131^I-low dose group (Fig. 7J, K; Additional file 5: Fig.S5C); the model achieved a total AUC of 0.72 in two classifiers (Fig. 7I) and showed good validation (*Z* > 0.05, Fig. 7J). *Escherichia-Shigella* was the only common genus in the two classifiers, which was sensitive to ^131^I therapy, consistent with the results in Fig. 3F. Besides, Model4 was aimed to distinguish between the Post-^131^I-2nd group and the Pre-^131^I-2nd group but could not be constructed as no factors were incorporated into models, indicating no significant differences between the two groups.

## Discussion

**Fig. 8:**
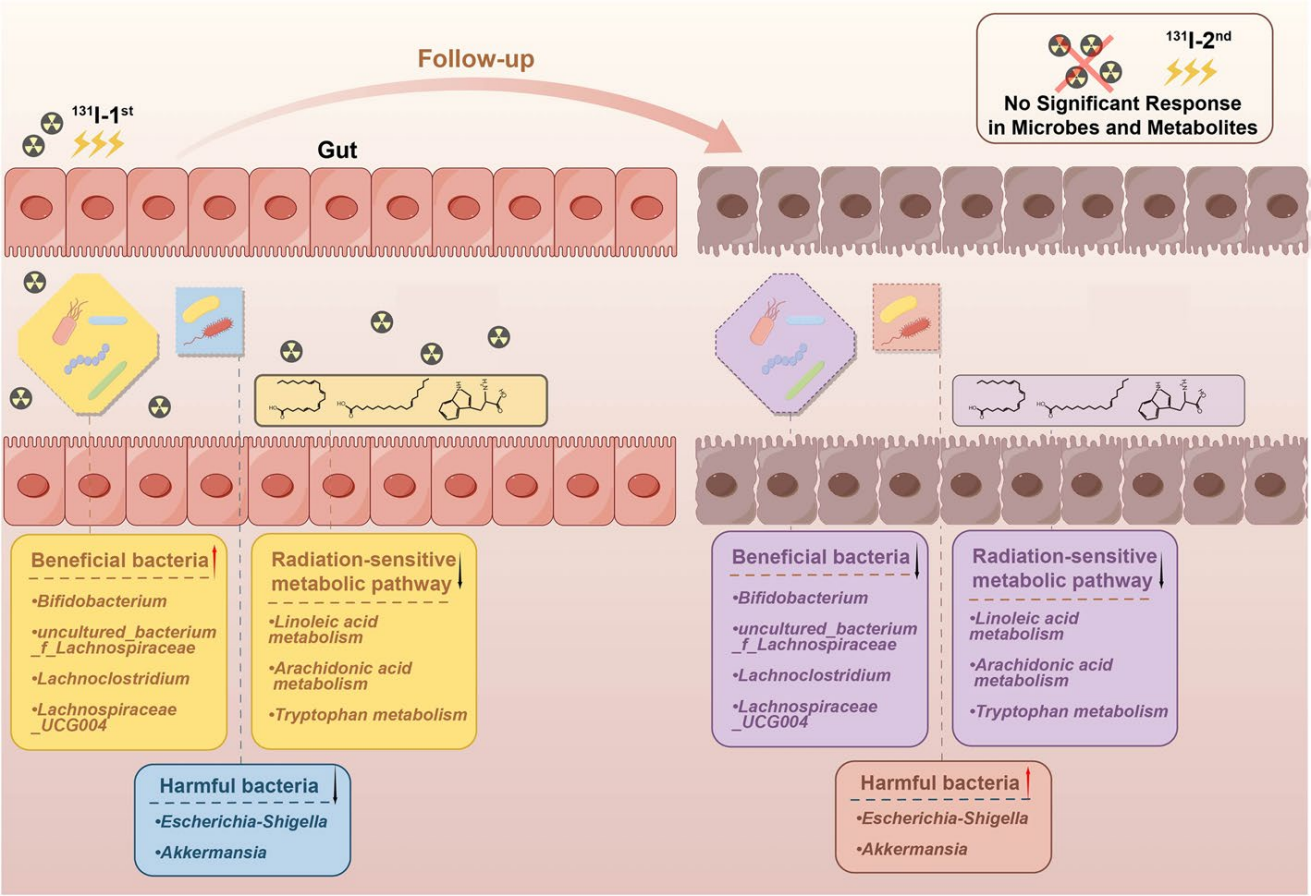
Putative mechanism of disrupted gut microbiome accompanied with fecal metabolites in DTC patients undertaking twice ^131^I therapy. Fluctuations of intestinal flora and fecal radiation-sensitive metabolites in the ^131^I treatment progress, which might reflect and regulate enteric microecology disrupted by radionuclide interruption

Recent research has revealed that gut microbiomes and metabolism are significantly altered by IR [5–10, 24–26]. However, few studies have focused on the effects of IR from radionuclides [4, 21, 27, 28]. According to a recent position article by the European Association of Nuclear Medicine [29], radiotherapy cannot be extrapolated to nuclear medicine directly, due to differences in dose-rate effects, linear energy transfer, dosimetry, fractionation, duration of treatment delivery, range, and target volume [30–32]. Therefore, an integrated study of fecal microbe genomics and fecal metabolomes was performed in a DTC cohort of multiple ^131^I therapies. The fluctuations of intestinal flora and fecal metabolites were investigated in the ^131^I treatment process, which might reflect the disrupted enteric microecology by radionuclides (Fig. 8). The results indicated that some gut bacteria and fecal metabolites might serve as noninvasive biomarkers for acute and chronic responses of DTC patients under ^131^I treatment.

In the early response for ^131^I therapy (4 days), the findings suggested a decreased abundance of *Akkermansia* and *Escherichia-Shigella* and an increased abundance of *Bifidobacterium*, *Lachnoclostridium*, *uncultured_bacterium_f_Lachnospiraceae*, and *Lachnospiraceae_UCG004*. In the mouse model of radiation enteritis with 18 Gy irradiation, the richness of *Akkermansia* and *Escherichia-Shigella* after irradiation might contribute to radiation-induced pathogenesis [33, 34]. Regarding the ability to produce short-chain fatty acids (SCFA), *Bifidobacterium* and *Lachnospiraceae* are commonly recognized to play a beneficial role in gut health, further adopted in the treatment of gastrointestinal diseases induced by radiation [6, 35]. Our results indicated that ^131^I therapy (about 100 mCi) cleared harmful bacteria and preserved the beneficial bacteria in a short period of time. Additionally, our study identified the downregulated pathways of LA, ARA, and tryptophan metabolism after ^131^I therapy. Some metabolites involved in pathways of tryptophan have been found to significantly decrease the damage of radiation, such as 3-propionic acid [9], indole-3-carboxaldehyde [6], and kynurenic acid [6]. Moreover, increased production of LA, the precursor of ARA, strongly contributed to radiation resistance, with ARA-derived metabolite inhibitors exerting sensitizing effects, including aspirin [36, 37]. Wang’s research and our results further verified the radioprotective effect of supplementing ARA and LA [20, 21]. Based on these findings, all differential pathways in our study were hypothesized to be involved in radiation resistance, and inhibiting these radiation-sensitive metabolic pathways might result in radiation damage.

In the long-term response for ^131^I therapy (6 months), strikingly different results were found compared with the early response, including the deterioration of microbiome α diversity, with four beneficial bacteria reduced and two harmful bacteria improved; differentially expressed metabolites were downregulated and radiation-sensitive metabolic pathways were silenced; TC showed greater progression in the Pre-^131^I-2nd group compared to the Pre-^131^I-1st group, which was consistent with Zheng’s findings [4]. Gut microbiota might be a short-term protective factor against radionuclide toxicity due to their ability to self-regulate but cannot compensate over extended periods, leading to persistent metabolic damage. ^131^I therapy exerts a significant negative influence on gut microbiota and metabolism [4]. However, the alterations in bacteria or metabolites after the second ^131^I therapy with a higher dose were not as great as the first therapy, despite showing the same tendency. This indicated the limited ability of the body to regulate gut microbiota and metabolism against multiple radionuclide interventions. Gambale’s study also revealed that only a minority of cases benefit from the second ^131^I treatment [38]. Considering the non-significant response of intestinal ecology to the second ^131^I treatment, additional research should investigate the relations between the therapeutic effect of a second ^131^I and the regulation of gut microbiota and metabolism.

The integrated network analysis based on Spearman’s correlation revealed mutual interactions among the genera from two patterns and metabolic products from three radiation-sensitive metabolic pathways. In parallel, *Lachnoclostridium*, *Lachnospiraceae_UCG004*, *Escherichia-Shigella*, and LA-derived metabolites were the primary contributors to our integrated network, with high connection with inflammation factors across the ^131^I therapy. *Escherichia-Shigella* was reported to play a crucial role in proinflammatory responses [39]. *Lachnospiraceae* is a well-known SCFA producer, recognized as a regulator for intestinal inflammation [40, 41]. Metabolites in the LA metabolism pathway were found to have anti-inflammatory, anti-proliferative, and antithrombotic properties [22, 42]. Considering the proinflammatory and anti-inflammatory effects involved in these characterized bacteria and related metabolic pathways, and the potential function of LA pathway enrichment in repairing and promoting intestine recovery following ^131^I therapy [28], the replenishment of essential bacteria or metabolites were speculated to protect against radiation toxicity via inflammation resistance, needed to be investigated in future studies.

Finally, three diagnostic models were established, including key microbiota and metabolites for indicating the early (Model1), long-term (Model2), and dose-dependent (Model3) responses for ^131^I therapy by two classifiers. The reliability and accuracy of the three models were proved by the similar factors involved in each model by different classifiers and by their corresponding validation cohort. Model1 contains more than one-third of the factors of characterized bacteria and metabolites and showed a high AUC value, indicating the great discriminative ability of the model and the significant alterations in intestinal ecology in the early response to ^131^I therapy. Fewer factors were included in both Model2 and Model3 compared to Model1, which might be attributed to the lower number of patients in the analyzed cohorts. Nevertheless, the metabolites in the LA and ARA pathways were identified (PC(22:2(13Z,16Z)/14:0) and delta-12-Prostaglandin J2) as biomarkers for distinguishing the chronic response of ^131^I therapy, as opposed to the minor differences of clinical factors between DTC and Pre-^131^I-2nd groups. As the only common bacteria observed in Model3, Escherichia-Shigellas showed strong radiosensitivity under ^131^I therapy and might be recognized as an independent risk factor for screening the effective treatment dose with radionuclides in future studies.

Nevertheless, the limitations of the current study should be acknowledged. First, despite the integrated analyses of microbiomics and metabolomics, our study lacks novelty as the role of gut microbiota in response to ^131^I treatment in DTC patients has already been investigated. Second, only 16S rRNA gene sequencing and fecal metabolomic analyses were performed, and metagenomics and serum metabolomics are necessary for further investigation and functional analysis. Finally, the relationship between metabolites in radiation-sensitive metabolic pathways and other microbiota under ^131^I therapy remains unclear. Additional experimental verification might be required to further corroborate our findings.

## Conclusions

Considerable changes in the gut microbes were identified with metabolic alterations in LA, ARA, and tryptophan metabolism in DTC patients during multiple ^131^I therapy courses. Additionally, the cross-interactions among characterized bacteria and metabolites from three radiation-sensitive metabolic pathways demonstrated a close association with different stages of ^131^I treatment. Moreover, the early and long-term ^131^I treatment-related changes in gut microbiota combined with fecal metabolites might represent a noninvasive diagnostic method for radionuclide damage.

### Supplementary Information

Additional file 1: Fig.S1 Features of gut bacterial community composition and structure. Α diversity analysis in Simpson (A) and Shannon (B) indices among four groups. (C) Ratio of *Firmicutes*/*Bacteroidetes* (F/B) among four groups. * *p* < 0.05. DTC, differentiated thyroid cancer; Pre-^131^I-1st group, patients with DTC before the first ^131^I therapy; Post-^131^I-1st group, patients with DTC after the first ^131^I therapy; Pre-^131^I-2nd group, patients with DTC before the second ^131^I therapy; Post-^131^I-2nd group, patients with DTC after the second ^131^I therapy.

Additional file 2: Fig.S2 Alterations in microbial genus compositions at different stages of DTC with ^131^I therapy. Wilcoxon rank-sum test between Post- and Pre-^131^I-1st (A), Pre-^131^I-2nd and Pre-^131^I-1st (B), Post- and Pre-^131^I-2nd (C) groups. (D) Abundance of *g_Akkermansia* between low (< 150 mCi) and high (≥ 150 mCi) dose of^131^I therapy. * *p* < 0.05. DTC, differentiated thyroid cancer; Pre-^131^I-1st group, patients with DTC before the first ^131^I therapy; Post-^131^I-1st group, patients with DTC after the first ^131^I therapy; Pre-^131^I-2nd group, patients with DTC before the second ^131^I therapy; Post-^131^I-2nd group, patients with DTC after the second ^131^I therapy.

Additional file 3: Fig.S3 Linoleic acid as the potential radioprotectants under ^131^I therapy. (A) Schematic of linoleic acid (LA) treatment under 2 mCi ^131^I therapy. (B) Spleens stained with hematoxylin and eosin (H&E) (× 200 magnification). (C) The small intestines stained with H&E (× 200 magnification, broken intestinal epithelium, black arrow), (D) alcian blue/periodic acid-schiff (AB-PAS) (× 200 magnification, goblet cells, black arrow) and (E, F) immunohistochemistry (IHC) (× 200 magnification, stained with antibodies, black arrow). WP, white pulp.

Additional file 4: Supplemental Methods. Methods for the supplementation of linoleic acid in mice undergoing ^131^I therapy.

Additional file 5: Fig.S4 Disrupted gut microbiota interactions with fecal metabolism and clinical factors associated with DTC patients under ^131^I therapy. Spearman correlation analyses among six charactered genera (A) and 11 radiation-sensitive metabolites(B). (C) Spearman correlation analyses between six charactered genera and 11 radiation-sensitive metabolites.* *p* < 0.05,** *p* < 0.01,*** *p* < 0.001.

Additional file 6: Fig.S5 Predictive models combined with microbiome and metabolism for response to ^131^I therapy. The cross-validation process was repeated five times in random forest model of Model1 (A), Model2 (B) and Model3 (C).

## Data Availability

The original data presented in this study are available from the corresponding author upon reasonable request.

## Abbreviations

^131^I-1^st^: The first ^131^I therapy
^131^I-2^nd^: The second ^131^I therapy
ARA: Arachidonic acid
AUC: Area under the curve
BMI: Body mass index
CRP: C-reactive protein
DBI: Direct bilirubin
DTC: Differentiated thyroid cancer
F/B: *Firmicutes*/*Bacteroidetes*
FDR: False discovery rate
Gran: Neutrophil
Hb: Hemoglobin
HDL: High-density lipoprotein
IL-1: Interleukin 1
IL-2: Interleukin 2 receptor
IL-6: Interleukin 6
IR: Ionizing radiation
KEGG: Kyoto Encyclopedia of Genes and Genomes
LA: Linoleic acid
LDL: Low-density lipoprotein
LEfSe: Linear discriminate analysis effect size
Lymph: Lymphocyte
OTU: Operational taxonomic unit
PCA: Principal components analysis
PCoA: Principal coordinates analysis
PLS-DA: Partial least square regressions-discriminant analysis
PLT: Platelet
Post: -^131^I-1^st^After the first ^131^I therapy
Post-^131^I-1st/2^nd^: After the second ^131^I therapy
Pre-^131^I-1^st^: Before the first ^131^I therapy
Pre-^131^I-2^nd^: Before the second ^131^I therapy
RBC: Red blood cell
RDA: Redundancy analysis
RF: Random forest
ROC: Receiver operating characteristic
SCFA: Short-chain fatty acids
TBI: Total bilirubin
TC: Thyroid cancer
TCh: Total cholesterol
Tg: Thyroglobulin
TG: Triglyceride
TgAb: Thyroglobulin antibodies
TNFα: Tumor necrosis factor alpha
TSH: Thyrotropin
WBC: White blood cell
