# Supplementary figures and images for: Early and long-term responses of intestinal microbiota and metabolites to 131I treatment in differentiated thyroid cancer patients
Source: BMC Med. 2024 Jul 18;22:300. doi: 10.1186/s12916-024-03528-3 (PMC11256643; doi:10.1186/s12916-024-03528-3)

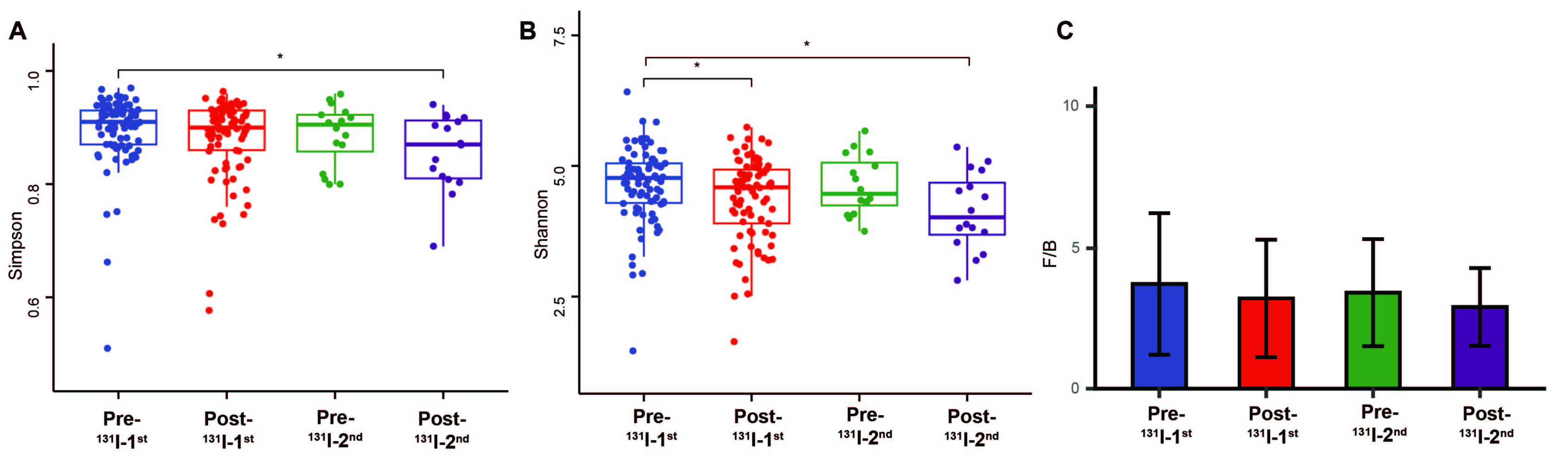

Supplement: Supplementary file 1 — Additional file 1: Fig.S1 Features of gut bacterial community composition and structure. Α diversity analysis in Simpson (A) and Shannon (B) indices among four groups. (C) Ratio of Firmicutes/Bacteroidetes (F/B) among four groups. * p < 0.05. DTC, differentiated thyroid cancer; Pre-131I-1st group, patients with DTC before the first 131I therapy; Post-131I-1st group, patients with DTC after the first 131I therapy; Pre-131I-2nd group, patients with DTC before the second 131I therapy; Post-131I-2nd group, patients with DTC after the second 131I therapy. [file 12916_2024_3528_MOESM1_ESM.jpg]

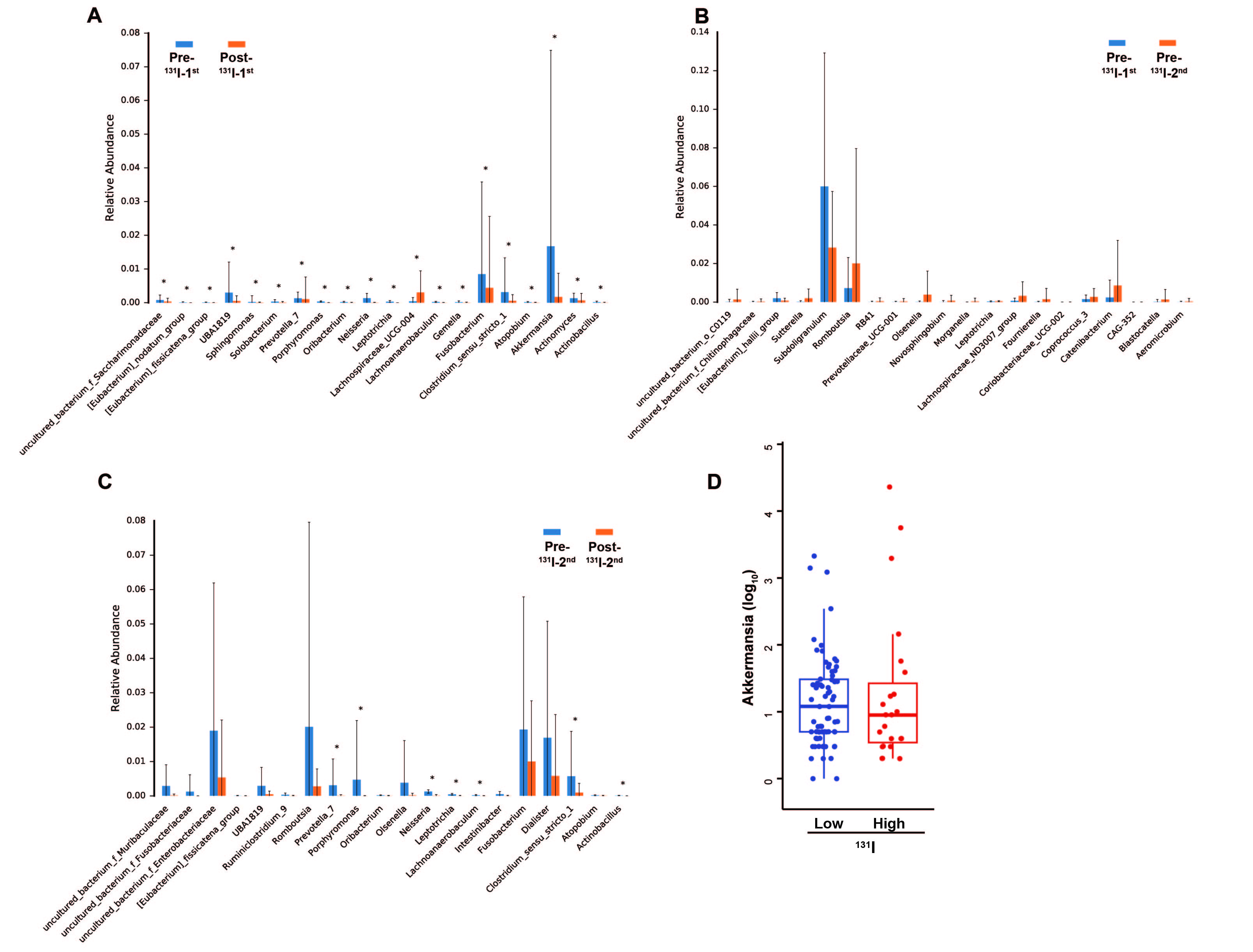

Supplement: Supplementary file 2 — Additional file 2: Fig.S2 Alterations in microbial genus compositions at different stages of DTC with 131I therapy. Wilcoxon rank-sum test between Post- and Pre-131I-1st (A), Pre-131I-2nd and Pre-131I-1st (B), Post- and Pre-131I-2nd (C) groups. (D) Abundance of g_Akkermansia between low (< 150 mCi) and high (≥ 150 mCi) dose of131I therapy. * p < 0.05. DTC, differentiated thyroid cancer; Pre-131I-1st group, patients with DTC before the first 131I therapy; Post-131I-1st group, patients with DTC after the first 131I therapy; Pre-131I-2nd group, patients with DTC before the second 131I therapy; Post-131I-2nd group, patients with DTC after the second 131I therapy. [file 12916_2024_3528_MOESM2_ESM.jpg]

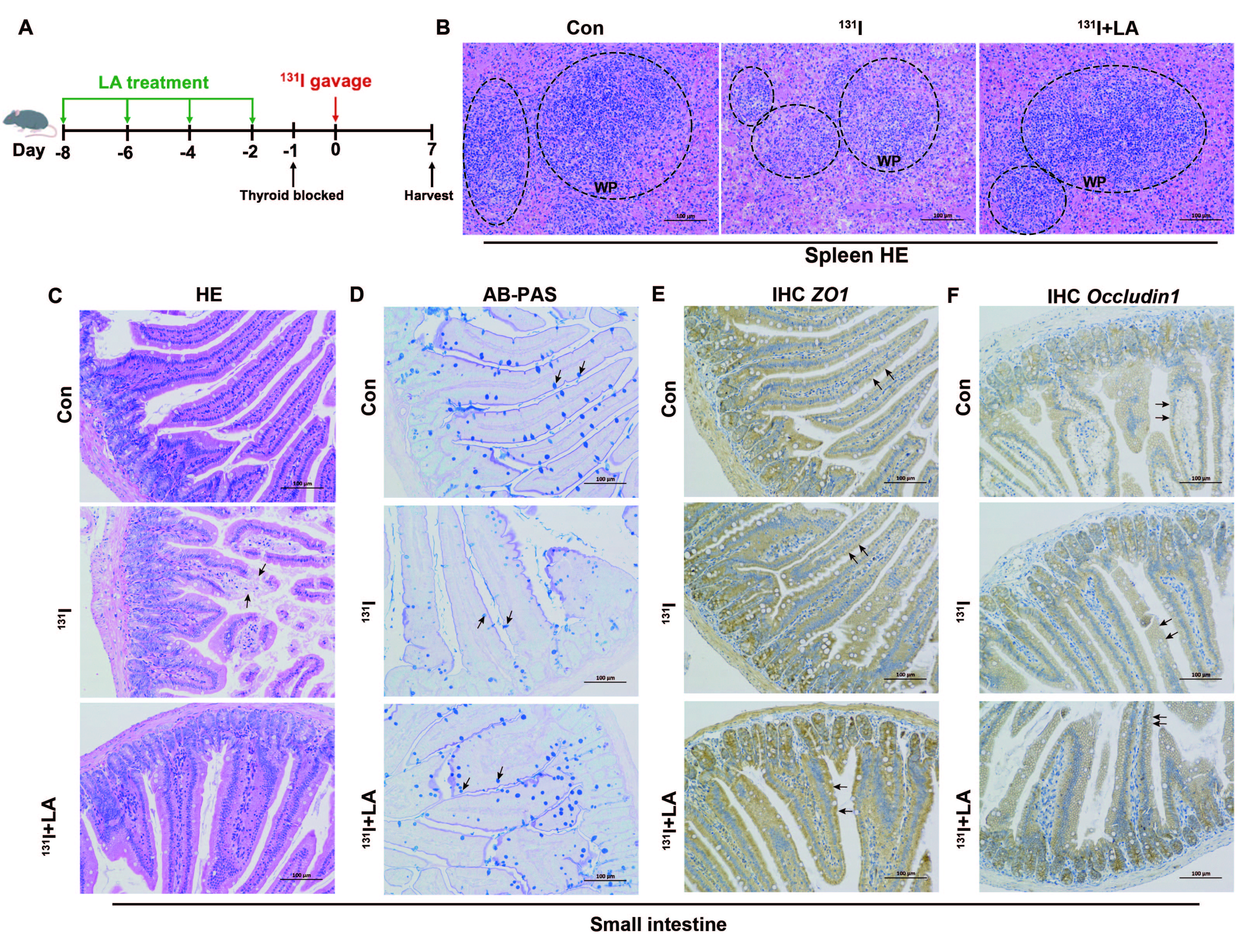

Supplement: Supplementary file 3 — Additional file 3: Fig.S3 Linoleic acid as the potential radioprotectants under 131I therapy. (A) Schematic of linoleic acid (LA) treatment under 2 mCi 131I therapy. (B) Spleens stained with hematoxylin and eosin (H&E) (× 200 magnification). (C) The small intestines stained with H&E (× 200 magnification, broken intestinal epithelium, black arrow), (D) alcian blue/periodic acid-schiff (AB-PAS) (× 200 magnification, goblet cells, black arrow) and (E, F) immunohistochemistry (IHC) (× 200 magnification, stained with antibodies, black arrow). WP, white pulp. [file 12916_2024_3528_MOESM3_ESM.jpg]

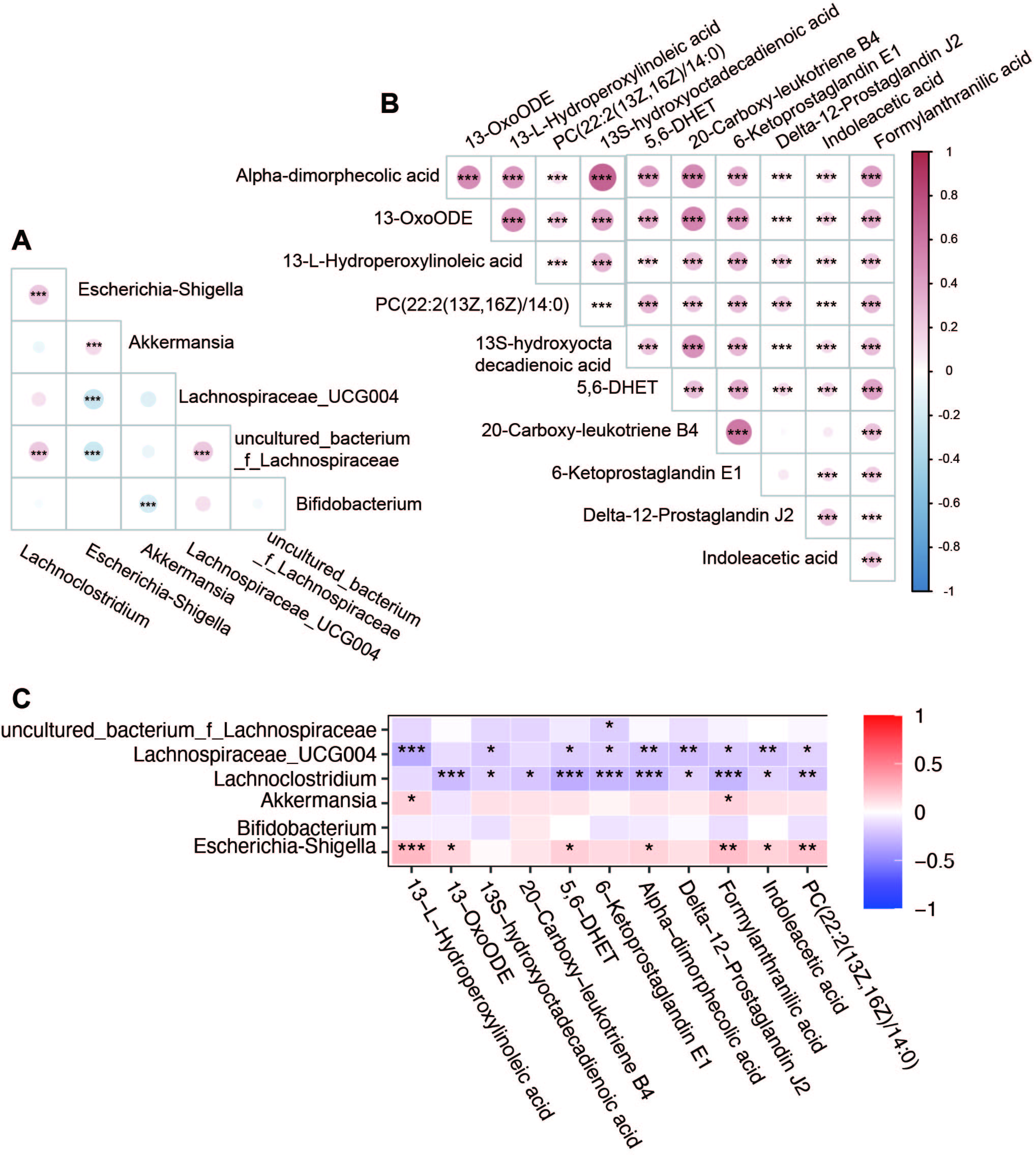

Supplement: Supplementary file 5 — Additional file 5: Fig.S4 Disrupted gut microbiota interactions with fecal metabolism and clinical factors associated with DTC patients under 131I therapy. Spearman correlation analyses among six charactered genera (A) and 11 radiation-sensitive metabolites(B). (C) Spearman correlation analyses between six charactered genera and 11 radiation-sensitive metabolites.* p < 0.05,** p < 0.01,*** p < 0.001. [file 12916_2024_3528_MOESM5_ESM.jpg]

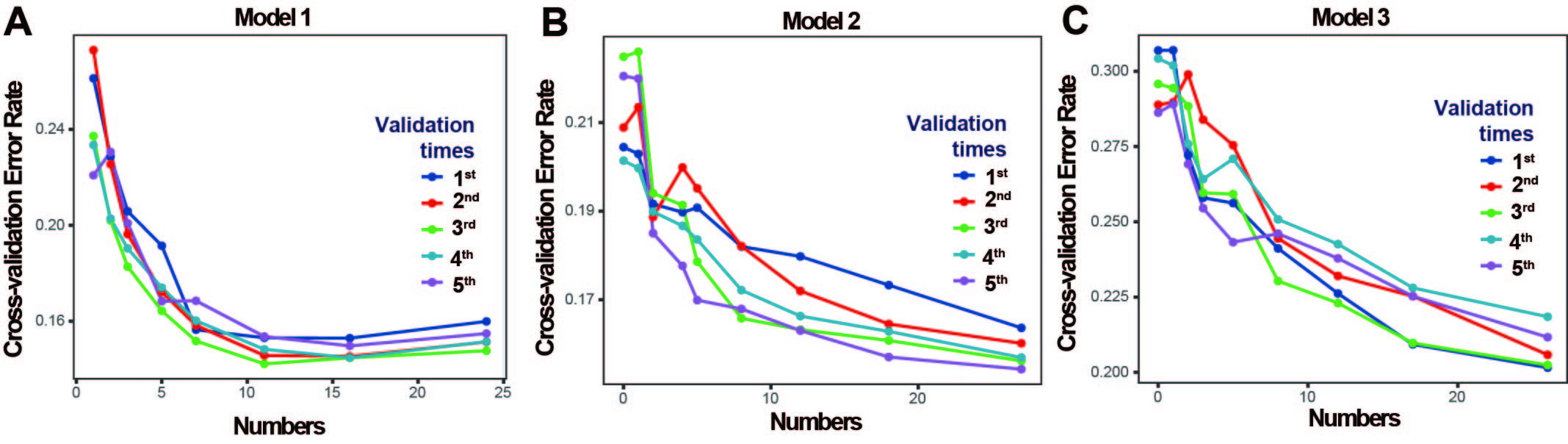

Supplement: Supplementary file 6 — Additional file 6: Fig.S5 Predictive models combined with microbiome and metabolism for response to 131I therapy. The cross-validation process was repeated five times in random forest model of Model1 (A), Model2 (B) and Model3 (C). [file 12916_2024_3528_MOESM6_ESM.jpg]
