# Supplementary material for: Early and long-term responses of intestinal microbiota and metabolites to 131I treatment in differentiated thyroid cancer patients
Source: BMC Med. 2024 Jul 18;22:300. doi: 10.1186/s12916-024-03528-3 (PMC11256643; doi:10.1186/s12916-024-03528-3)
Supplement: Supplementary file 4 — Additional file 4: Supplemental Methods. Methods for the supplementation of linoleic acid in mice undergoing 131I therapy. [file 12916_2024_3528_MOESM4_ESM.docx]

**Supplemental Methods**

***Mice***

6-8 weeks old male C57BL/6J mice and were purchased from the Beijing Vital River Laboratory Animal Technology Co., Ltd. (Beijing, China). The mice were housed in a specific pathogen-free (SPF) animal facility. Mice were kept in a controlled environment (22 ℃ ± 2 ℃, 60 % ± 5 % relative humidity, and 12 h light/dark cycle) and provided with a standard diet and water. All animal experiments were approved by the Animal Ethics Committee of Shanghai Tenth People’s Hospital (SHDSYY-2021-2801), which complied with the Guide for the Care and Use of Laboratory Animals.

***Experimental Treatments***

All mice were acclimatized for one week and randomly assigned to different groups with corresponding treatments (**Fig.S3A**): (1) Con group: healthy 6- 8 weeks old C57BL/6 mice. (2) ^131^I group: Mice were exposed to 2 mCi ^131^I oral administration (fed with 3% NaI water to block thyroid gland 24h before) [1, 2]. (3) ^131^I + LA group: Mice were treated with linoleic acid (LA) (Sigma, #8.43483)[3] 50 mg/kg through oral gavage, dissolved in sterile water mixed with an ultrasound shaker for 5 minutes in 0.2 ml volume per mouse from eight days to two days before ^131^I administration (interventions on alternate days). All animals were sacrificed one week after the ^131^I administration.

***Histopathology***

Spleens and small intestines were collected and fixed in 10% neutral-buffered formalin. Then, 4 µm sections of paraffin-embedded samples were stained with hematoxylin and eosin (H&E, Sigma-Aldrich). Paraffin-embedded sections were dewaxed in xylene and ethanol before staining with alcian blue/periodic acid-schiff (AB-PAS) C. Subsequently, the sections were washed with distilled water, acidified with AB-PAS B, and stained with AB-PAS A. Finally, the sections were washed with distilled water. In this study, samples were scored semi-quantitatively by a board-certified pathologist who was blinded to the experimental conditions. We performed immunohistochemistry (IHC) using commercially available antibodies. *ZO1* and *occludin* antibodies were purchased from Abcam (Cambridge, MA, USA).

**Reference**

1. Smit JW, Schröder-van der Elst JP, Karperien M, Que I, Stokkel M, van der Heide D, et al. Iodide kinetics and experimental (131)I therapy in a xenotransplanted human sodium-iodide symporter-transfected human follicular thyroid carcinoma cell line. J Clin Endocrinol Metab. 2002;87 3:1247-53. doi:10.1210/jcem.87.3.8307.

2. Zhao S, Shi D, Su C, Jiang W, Zhang C, Liang T, et al. IL-27Rα: A Novel Molecular Imaging Marker for Allograft Rejection. Int J Mol Sci. 2020;21 4 doi:10.3390/ijms21041315.

3. Martins JLR, Silva DM, Gomes EH, Fava SA, Carvalho MF, Macedo IYL, et al. Evaluation of Gastroprotective Activity of Linoleic Acid on Gastric Ulcer in a Mice Model. Curr Pharm Des. 2022;28 8:655-60. doi:10.2174/1381612826666200908144053.
